# Pilot implementation of the competence of Czech paramedics to administer sufentanil for the treatment of pain in acute trauma without consulting a physician: observational study

**DOI:** 10.1186/s12873-022-00622-8

**Published:** 2022-04-09

**Authors:** Metodej Renza, Roman Sykora, David Peran, Kristina Hricova, Nikola Brizgalova, Petra Bakurova, Miloš Kukacka

**Affiliations:** 1 Emergency Medical Services of Karlovy Vary Region, Karlovy Vary, Czech Republic; 2 Department of Anesthesia and Intensive Care, Third Faculty of Medicine, Charles University and FNKV University Hospital, Šrobárova 50, 100 34, Prague, Czech Republic; 3 Department of Nursing, Third Faculty of Medicine, Charles University, Prague, Czech Republic

**Keywords:** Prehospital care, Analgesia, Sufentanil, Paramedic, Simulation, Trauma

## Abstract

**Background:**

The use of intravenous opioids in the traumatic pain in pre-hospital care in the Czech Republic is based primarily on the indication of a physician. If the paramedic crew arrives at the site earlier or only on their own, analgesia is given after phone-call consultation with the physician or after his arrival at the site. The objective of this study was to evaluate the safety and efficacy of the indication and administration of sufentanil by paramedics in the treatment of pain in acute trauma adult patients without the physician’s control.

**Methods:**

Paramedics underwent voluntarily the simulation training aimed at administering intravenously sufentanil to treat pain in acute trauma in adults without physician’s indication. Subsequently, the adverse events and efficacy were monitored for a six-month period and compared in two groups: administration of sufentanil by paramedics without this competence, who further consulted the administration by telephone with physicians (group Consultation) and those with this competence (group Competence).

**Results:**

A total number of sufentanil administration in group Consultation was 88 and in group Competence 70. There was no respiratory arrest, bradypnea, or need for oxygen therapy reported in any of the study groups. The incidence of nausea was 3% in both groups – Consultation (*n* = 3) and in Competence (*n* = 2). Vomiting was not reported in the Consultation group and in 6% in the Competence group (*n* = 4). Intravenous antiemetic drugs were used in the Consultation group only in 1% (*n* = 1) and in the Competence group in 7% of patients (*n* = 5) (*p* < 0,05). In both groups there was observed a decrease in the pain numeric rating scale (Consultation: M =—3,2; SD = 1,2 points vs. Competence: M =—3,9; SD = 1,8 points).

**Conclusion:**

Intravenous administration of sufentanil by properly trained paramedics without consultation with a physician in acute trauma can be considered safe.

## Background

High quality and safe pain management is the goal of not only prehospital emergency care [1] provided by emergency medical services’ (EMS) crews. In terms of competencies, systems of providing pre-hospital emergency care differ. In many European countries, also in the Czech pre-hospital emergency care, the system is historically dependent on physicians [2, 3]. Competencies of paramedics are based on specific legal norms and education of healthcare professionals and differs all around the world [4, 5]. Paramedic’s crews in the Czech Republic do not have the competency to administer analgesic medication without direct supervision of or without phone-call consultation with an EMS physician [6]. It must be stated that shortage of physicians in the Czech EMS system due to personnel and economic reasons leads to an increasing emphasis on the competencies of paramedics. However, the Czech system of healthcare legislation allows the employer or organization to delegate certain competencies to paramedics within completely standard procedure in a defined situation (also known as Standard Operating Protocol). Among the growing number of competencies of Czech paramedics there is still a need to treat acute pain in acute traumatic injuries with opioids in the case of less serious cases where no ambulance crew with physician is dispatched to the scene [7]. At present, the absence of a physician in the ambulance crew on site leads to the need for a telephone consultation or a request for the arrival of a physician, which prolongs the time until effective analgesia and prolongs the patient’s suffering. In addition, during and after the telephone consultation, the EMS physician is not present on the scene to address any complications.

## Objectives

Primary objective of this study was to evaluate the safety and efficacy of indication and administration of the sufentanil in treatment of pain in acute trauma patients by paramedics without the physician’s control.

## Methods

Prior to the study a questionnaire survey focused on pain management in acute trauma among all paramedics of the EMS of Karlovy Vary Region was conducted (*n* = 115, return rate 81% (*n* = 95)). Based on main results of this survey the proposed competency to administer sufentanil by trained paramedics in acute trauma was identified as necessary for 80% (*n* = 76) of paramedics. Subsequently, this competence was determined as voluntary. A total of 39 paramedics signed up and completed training program to administer sufentanil in acute trauma. Age, gender, length of practice and level of medical education (whether university or higher professional school) were monitored in the group of paramedics who volunteered to acquire the competence. The training consisted of theoretical e-learning part (14 days prior the training) and one training session (4 h) based on medical simulations during August 2020. The training covered pharmacological and clinical information and specific indication criteria (case of acute traumatic pain without the presence of physician on site, adult patient without impaired consciousness, hemodynamic stability) and also the training of detection and management of complications, with special emphasis on respiratory depression and bag mask ventilation was trained. Sufentanil was titrated, based on clinical effect, by 5 µg up to 20 µg of maximal possible dose within this competence. The final verification of the competence of paramedics was evaluated by 3 instructors (anaesthesiologists working in EMS) during six simulation scenarios, hands-on station with bag mask ventilation and by final written exam focused on side effects, indication criteria and dose of sufentanil.

### Study design

This was a single centre, prospective, observational study with two monitored groups. The educational program, definition of competence and its implementation to internal standard of care was approved by the Medical Board of Emergency Medical Services of Karlovy Vary region on 22^nd^ of May 2020. The study protocol and conduction of the study was approved by Ethical Committee of Emergency Medical Services of Karlovy Vary Region registered with State Institute for Drug Control of the Czech Republic on 11^th^of September 2020 under ref. no. ZN/78/ZZSKVK/20. Informed consent was not required from patients with acute trauma pain. It was carried out within the framework of tacit consent after standard information about the planned procedure within the provision of pre-hospital emergency care according to the Czech legislation ((Health Services Act No. 2011, 372 (CZ)) [6]. The protocol of the trial was retrospectively registered at clinicaltrials.com (NCT04913402).

### Study location

The study was conducted at EMS of Karlovy Vary Region, Karlovy Vary, the Czech Republic during 6-month period from 11^th^ of September 2020 to 22^nd^ of March 2021. A total number of 20.406 patients were treated during the study period by crews of EMS of Karlovy Vary Region.

### Participants and interventions

All cases of administered sufentanil were checked from electronical patient documentation („ePaRe “ – part MZD, European Medical Distribution Ltd., Bratislava, Slovak Republic). Subsequently, only events which met eligibility criteria were included in analysis. Eligibility criteria consist of a) administration of sufentanil by paramedics on site without physical presence of physician in acute traumatic pain, b) adult patients (at least 18 years old), c) no impairment of consciousness (defined as Alert and Glasgow Coma Scale = 15), d) who is hemodynamically stable (defined by systolic blood pressure > 100 mmHg and without presence of bradycardia bellow 60 beats per minute). The two study groups were identified from eligible cases of patients with pain in acute trauma, who were given sufentanil 1) by paramedics in a routine way, after telephone consultation with an EMS physician (Consultation) and 2) who were given sufentanil by competent paramedics themselves (Competency). In the Competency group, the paramedics were allowed to administer sufentanil intravenously up to 20 µg. The recommended pain Numeric Rating Scale (NRS) score for consideration of sufentanil administration was above 4 points.

Baseline characteristics of both groups were obtained from electronical patient documentation: age, gender, NACA score (National Advisory Committee for Aeronautics), type of trauma (lower or upper limb, trauma of torso, head injury), dose of sufentanil and if there was fractional dose administration, and proportion of cases where additional non-opioid analgesia (paracetamol) was used.

### Outcome measures

To measure the safety and efficacy of sufentanil administration information were extracted from electronical patient documentation. Incidence of respiratory arrest (the need for bag mask ventilation); bradypnea (less than 10 breaths per minute); or need for oxygen therapy (defined as decrease of SpO_2_ under 92 percent). Then the frequency of complete NRS reporting (before sufentanil administration and at the handover) were determined. Other adverse effect of sufentanil administration (incidence of nausea and vomiting and need for intravenous antiemetic drug – thiethylperazin) was evaluated. Moreover, the heart rate, blood pressure, SpO_2_ and respiratory rate before sufentanil administration and at the handover were noted.

### Statistical methods

No sample size was calculated prior to the study but the period of half a year was set for evaluation. Due to the character of observations and predefined groups of paramedics with and without competency no randomization or blinding was used. Baseline characteristics and outcomes among study groups were tested by t-test for ordinal and Chi-square test for nominal variables. Statistical software STATISTICA 7.0 (StatSoft, Inc., Tulsa, Oklahoma, USA) was used for statistical analysis and calculations.

## Results

**Fig. 1:**
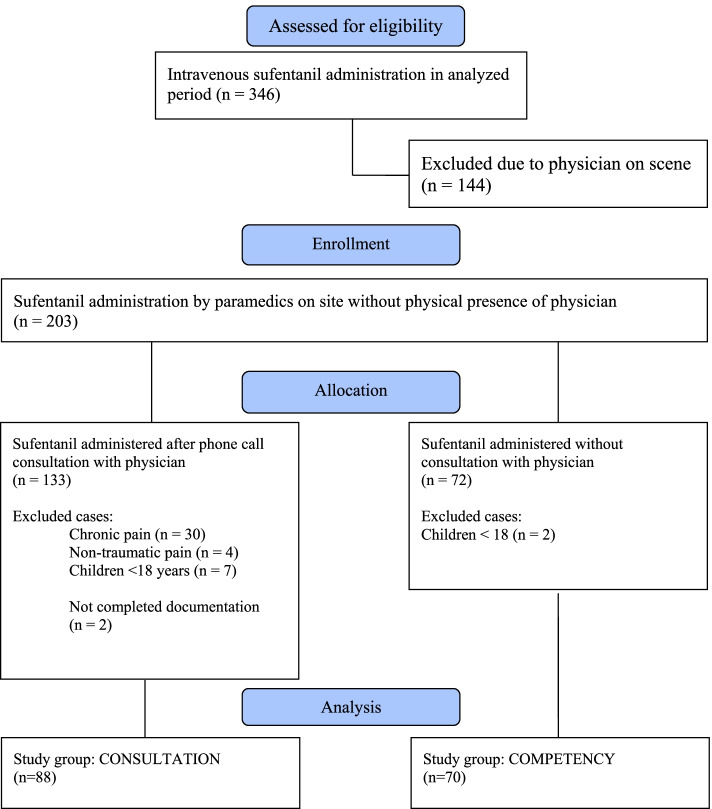
Flow diagram

The selection of eligible and description of excluded cases is presented in the flow diagram (Fig. 1). Baseline characteristics of the paramedics with competency are compared with those who did not volunteer in Table 1. A total number of sufentanil administered intravenously to patients with acute trauma by paramedics after phone call consultation with EMS physician (group Consultation) was 88 and by paramedics with competency without any consultation (group Competence) was 70.

**Table 1:** Baseline statistics of group of paramedics without and with competence

	CONSULTATION (*n* = 76)	COMPETENCE (*n* = 39)	
Age (years)	44.7 (9.6)	44.1 (8.5)	NS
Gender (women)	63%	59%	NS
University education	28%	44%	NS*
Length of praxis (years)	21.5 (10.0)	20.4 (8.9)	NS

Data are presented as mean and standard deviation or as number and percentage

*NS* Not Significant

^*^ *p* = 0.085

The baseline characteristics of both groups including the spectrum of injuries and pharmacotherapy is in detail described in Table 2. The dose of intravenously administered sufentanil was almost identical in both groups (Consultation: M = 9.1; SD = 2.0 micrograms vs. Competence: M = 9.4; SD = 2.4 micrograms).

**Table 2:** Sufentanil administration baseline statistics

	CONSULTATION (*n* = 88)	COMPETENCE (*n* = 70)	
Age (years)	64,6 (19,7)	65,7 (20,0)	NS
Sex (women)	61 (69%)	39 (56%)	NS
NACA score	2,5 (0,5)	2,4 (0,5)	NS
Trauma of lower limb	57 (65%)	35 (50%)	NS^a^
Trauma of upper limb	23 (26%)	22 (31%)	
Trauma of torso	8 (9%)	13 (19%)	
Trauma of head	0	0	NS
Dose of sufentanil (µg)	9,1 (2,0)	9,4 (2,4)	NS
Fractional administration	24 (27%)	20 (29%)	NS
Additional non-opioid analgesia	12 (14%)	10 (13%)	NS

Data are presented as mean and standard deviation or as number and percentage

*NS* Not Significant, *NACA* National Advisory Committee for Aeronautics

^a^The 3 × 2 contingency table to calculate Chi square statistics was used

In terms of reporting the occurrence of adverse events (Table 3) there was no respiratory arrest or bradypnea reported in any of the study group as well as the need for oxygen therapy after sufentanil administration. The incidence of nausea was the same in both groups: Consultation; *n* = 3 (3%) vs Competence; *n* = 2 (3%). Vomiting after sufentanil administration was not reported in the Consultation group and despite the incidence of vomiting (6%) in the Competence group (*n* = 4) this result did not reach statistical significance. Similarly, intravenous antiemetic drugs were used less frequently in the consultation group only in 1% (*n* = 1) than in the Competence group in 7% of patients (*n* = 5) ((χ^2^(1, *N* = 158) = 3,85, *p* < 0,05).

**Table 3:** Adverse events and its treatment after intravenous sufentanil administration in trauma

	CONSULTATION (*n* = 88)	COMPETENCE (*n* = 70)	
Respiratory arrest	0	0	NS
Bradypnea	0	0	NS
Oxygen therapy after sufentanil administration	0	0	
Nausea	3 (3%)	2 (3%)	NS
Vomiting	0	4 (6%)	NS
Antiemetics administration	1 (1%)	5 (7%)	*P* < 0,05

Data are presented as number and percentage. *NS* Not Significant

In both groups there was a decrease of pain in the NRS (Consultation: M =—3,2; SD = 1,2 points vs. Competence: M =—3,9; SD = 1,8 points) without a statistically significant difference between the groups: t(81) = -1,58. *p* = 0,059. Complete NRS reporting was significantly more frequently reported in the Competency group in 86% (*n* = 60) compared to 26% (*n* = 23) in the Consultation group (χ^2^(1, *N* = 158) = 47,35, *p* < 0,0001). From such disproportionately reported data, a significant difference in the NRS was evident between Consultation (M =—6,4; SD = 1,5) and Competency group (M =—7,9; SD = 1,4): t(81) = -4,05, *p* < 0,05 as presented in Table 4.

**Table 4:** Numeric rating scale (NRS) details

	CONSULTATION (*n* = 88)	COMPETENCE (*n* = 70)	
Complete report of NRS	23 (26%)	60 (86%)	*P* < 0.05
NRS reduction (points)	-3.2 (1.2)	-3.9 (1.8)	NS
Initial NRS (points)	6.4 (1.5)	7.9 (1.4)	*P* < 0.05

Data are presented as mean and standard deviation or as number and percentage

NS Not Significant, *NRS* pain Numeric Rating Scale

Any significant effect of sufentanil administration on systolic and diastolic blood pressure, heart rate, peripheral oxygen saturation and respiratory rate was not observed and its differences in patients of both groups before administration of sufentanil and on handover (Table 5) and the values between both study groups did not differ significantly as well.

**Table 5:** Effect of intravenous sufentanil administration in trauma on physiological parameters

	CONSULTATION (*n* = 88)		COMPETENCE (*n* = 70)		
	On scene	Handover	On scene	Handover	
Systolic blood pressure (mmHg)	145.5 (24.1)	142.9 (20.7)	142.2 (20.8)	138.9 (16.5)	NS
Systolic blood pressure difference (mmHg)		-2.7 (13.2)		-3.3 (11.2)	NS
Diastolic blood pressure (mmHg)	78.3 (15.2)	78.1 (10.9)	79.8 (11.6)	77.5 (10.3)	NS
Diastolic blood pressure difference (mmHg)		-0.2 (11.2)		-2.2 (7.6)	NS
Heart rate (bpm)	85.9 (13.2)	84.5 (13.4)	86.0 (15.9)	83.2 (14.0)	NS
Heart rate difference (bpm)		-1.4 (8.0)		-2.9 (7.8)	NS
SpO_2_ (%)	96.9 (9.8)	96.7 (2.3)	97.1 (1.7)	96.8 (1.4)	NS
SpO_2_ difference (%)		-0.2 (1.8)		-0.3 (1.7)	NS
Respiratory rate (breaths per minute)	14.4 (1.5)	14.1 (1.5)	15.6 (2.6)	14.6 (1.6)	NS
Respiratory rate difference (breaths per minute)		-0.3 (1.1)		-1.1 (2.0)	NS

Data are presented as mean and standard deviation

*NS* Not Significant, *bpm* beats per minute, *SpO*_*2*_ Oxygen Saturation

## Discussion

This study focused on the creation of a new competence for paramedics in a physician-based system of prehospital emergency care [2], when paramedics usually do not have the competence to administer opioid analgesics. On the other hand, due to the urgency of emergency calls, paramedic’s crews are often sent to cases of less serious trauma on their own without a doctor crew [7]. These trauma patients are obviously in pain and paramedics should either call a doctor on the scene or consult him for analgesia administration. From the available opioids provided in pre-hospital care in the Karlovy Vary region of the Czech Republic, the most frequently used opioid sufentanil was selected. Although sufentanil is a very potent opioid [8] it has been confirmed by this study that when administered within a clearly defined indication and by well-trained paramedics, it is an effective and safe alternative to administration by telephone consultation with EMS physician. In addition, the authors believe that the paramedics training to solve complications after administration of sufentanil is beneficial not only for this particular competence, but it can also be used in other situations where the doctor prescribes by phone and is not present on the scene to solve possible complications.

In the results, there was recorded relatively few side effects, which is probably the result of strict indication criteria. Such a criteria were deliberately set very harshly to ensure that sufentanil was safe and that training was adequate. Reducing the number of phone-call consultations with an EMS physician leads to a lower burden on paramedics and physicians. This study verified that it is possible to assign other competencies based on simulation training with verification of knowledge and skills. In this study, the clinical benefit in reducing the time of patient’s suffering and pain can be expected. Unfortunately, the exact time of administration of sufentanil and the possible reduction in time to its administration without consulting a physician were not monitored in this study due to operative and ethical reasons.

This single centre observational study has several limitations. One of them is that new competence was given to paramedics who voluntarily underwent training. This voluntariness could cause the bias of this study. Motivated paramedics have usually better performance than unmotivated. A very interesting finding was that among the paramedics who acquired the competence to administer sufentanil voluntarily, no difference was observed in any of the following parameters (age, gender, length of practice) compared to those paramedics who did not want the competence. However, the higher percentage of university education among paramedics who acquired competence did not gain statistical significance, probably due to the size of the sample and its disproportion.

In addition, the analysis of the results was from a relatively short period of time when it was burdened with other special conditions, especially COVID-19 patients. Due to waves of COVID-19, quarantine measures and reduced population movements, trauma in pre-hospital care has decreased.

It is certainly worth mentioning the difference in NRS reporting between groups. The study was conducted as a prospective observational study. The control group performed routine work (blinded) and only trained rescuers had to respect the new standard of care, which includes the obligation to report to the NRS when considering opioid administration [9] based on new competence. This may partly explain the difference in complete NRS reporting before and after administration of sufentanil. Likewise, paramedics who should have consulted physicians may tend to underestimate NRS and even monitor for side effects. This statement can be based on the lower need for therapeutic administration of antiemetics in the control group. From these data it is possible to conclude that increasing the level of competencies of paramedics or education [10] can lead to an increase in the quality and safety of care provided thanks to a higher level of responsibility and motivation, which leads to more careful examination of patients, better focus on their needs and in the end also better medical documentation. At the same time, a group with competencies can be perceived as more proactive, as well as with a tendency towards better reporting and earlier treatment using their other competencies.

In general, the treatment of pain by opioids is still open area in emergency medicine and especially in the pre-hospital setting [11, 12]. This study focused on the administration of intravenous sufentanil in less severe traumatic injuries. So far it seems to be the first study addresses the use of intravenous sufentanil in acute trauma by paramedics without EMS physician consultation. Most studies focus on the administration of fentanyl or morphine [13], ketamine [14, 15], combination of fentanyl and ketamine [16] or on another route of administration (e.g. intranasal or transmucosal) [17–19]. Our study does not address the use of opiates in children or other medical conditions (e.g. myocardial infarction) as it was presented in other studies [13, 20].

And at the same time, this study is in agreement with other studies from similar health care systems and confirms that it is possible to entrust prehospital analgesia to trained paramedics [14]. In the end we must mention that further validation by randomized controlled trial would be beneficial.

## Conclusion

Intravenous administration of sufentanil by paramedics alone without consultation with a physician (in physician-based healthcare systems) in acute adult trauma can be considered safe within the scope of trained and established competence and in compliance with the indication criteria.

## Data Availability

The data generated, analysed and used during the current study are available from the corresponding author on reasonable request. The data that support the findings of this study are available from the Emergency Medical Services of Karlovy Vary region, but restrictions apply to the availability of these data, which were used under license for the current study, and so are not publicly available. Data are however available from the authors upon reasonable request and with permission of Emergency Medical Services of Karlovy Vary region.

## Abbreviations

EMS: Emergency Medical Service
NACA: National Advisory Committee for Aeronautics
NRS: Numeric Rating Scale
NS: Not Significant
mmHg: Millimeters of Mercury
bpm: Beats Per Minute
µg: Micrograms
